# The effect of non-pharmacologic strategies on prevention or management of intensive care unit delirium: a systematic review

**DOI:** 10.12688/f1000research.25769.2

**Published:** 2021-09-24

**Authors:** Julie S Cupka, Haleh Hashemighouchani, Jessica Lipori, Matthew M. Ruppert, Ria Bhaskar, Tezcan Ozrazgat-Baslanti, Parisa Rashidi, Azra Bihorac

**Affiliations:** 1 Department of Medicine, University of Florida, Gainesville, FL, 32608, USA; 2 Precision and Intelligent Systems in Medicine (PrismaP), University of Florida, Gainesville, FL, 32608, USA; 3 Department of Biomedical Engineering, University of Florida, Gainesville, FL, 32608, USA

**Keywords:** critical care, delirium, intensive care, non-pharmacologic, systematic review

## Abstract

**Background:** Post-operative delirium is a common complication among adult patients in the intensive care unit. Current literature does not support the use of pharmacologic measures to manage this condition, and several studies explore the potential for the use of non-pharmacologic methods such as early mobility plans or environmental modifications. The aim of this systematic review is to examine and report on recently available literature evaluating the relationship between non-pharmacologic management strategies and the reduction of delirium in the intensive care unit.

**Methods:** Six major research databases were systematically searched for articles analyzing the efficacy of non-pharmacologic delirium interventions in the past five years. Search results were restricted to adult human patients aged 18 years or older in the intensive care unit setting, excluding terminally ill subjects and withdrawal-related delirium. Following title, abstract, and full text review, 27 articles fulfilled the inclusion criteria and are included in this report.

**Results:** The 27 reviewed articles consist of 12 interventions with a single-component investigational approach, and 15 with multi-component bundled protocols. Delirium incidence was the most commonly assessed outcome followed by duration. Family visitation was the most effective individual intervention while mobility interventions were the least effective. Two of the three family studies significantly reduced delirium incidence, while one in five mobility studies did the same. Multi-component bundle approaches were the most effective of all; of the reviewed studies, eight of 11 bundles significantly improved delirium incidence and seven of eight bundles decreased the duration of delirium.

**Conclusions:** Multi-component, bundled interventions were more effective at managing intensive care unit delirium than those utilizing an approach with a single interventional element. Although better management of this condition suggests a decrease in resource burden and improvement in patient outcomes, comparative research should be performed to identify the importance of specific bundle elements.

## Introduction

Delirium is a multifactorial, acute, confusional state characterized by the disturbance of consciousness and cognition; it is particularly common in the intensive care unit (ICU) with incidence ranging from 19 to 87% with especially high rates in mechanically ventilated patients ^1– 3^. ICU delirium is associated with adverse outcomes including increased mortality, prolonged mechanical ventilation and hospitalization, increased risk of cognitive dysfunction after discharge, and increased cost of care ^4– 7^.

While the pathophysiology of delirium is not well understood, there are multiple factors associated with an increased risk for developing delirium including age, neurologic or psychological disorders, polypharmacy, medications, and sensory impairment ^8– 11^. Modifiable environmental risk factors including immobilization, use of restraints, isolation, and levels of environmental light and sound are also considered risk factors for the development of delirium in the ICU ^8, 12^.

The morbidity associated with delirium as well as the multitude of delirium risk factors present in the ICU make delirium prevention and management strategies essential. These strategies have included pharmacological, non-pharmacological, and multicomponent interventions with the aim of decreasing the incidence and duration of delirium. Research into pharmacological interventions has focused on haloperidol and dexmedetomidine, though there has also been limited research into the effects of ramelteon, melatonin, and ziprasidone ^13– 16^. Despite continued research, current literature does not support the use of anti-psychotic agents, benzodiazepines, or melatonin in the management of delirium ^13, 17^.

Given the lack of evidence supporting pharmacological measures, further research into the efficacy of non-pharmacologic interventions such as early mobilization, environmental modifications, or management bundles is crucial. The implementation of effective delirium management shows promise in decreasing morbidity, mortality, length of stay, and resource burden in the ICU setting. In terms of the PICOS framework (Population, Interventions, Comparisons, Outcomes, Study Design) ^18^, our systematic review aims to address the effects of any non-pharmacologic prevention or management strategy on the incidence, prevalence, duration, or severity of delirium in critically ill adult patients compared to control patients, with no restrictions on study design.

## Methods

### Search strategy and data extraction

The Preferred Reporting Items for Systematic Reviews and Meta-Analyses (PRISMA) guidelines were followed in this review and included as *Reporting guidelines* ^19, 20^. The electronic databases of PubMed, Embase, Cochrane Central, Web of Science, The Cumulative Index to Nursing and Allied Health Literature (CINAHL), and ClinicalTrials.gov were systematically searched on May 15, 2019 for articles concerning non-pharmacologic treatments for delirium in the ICU. Search terms were tailored to each database in order to best utilize the individual subject headings, keywords, and medical subject headings (MeSH) terms included in the individual databases. A full list of search terms is shown in the *Extended data* ^20^.

**Figure 1.**
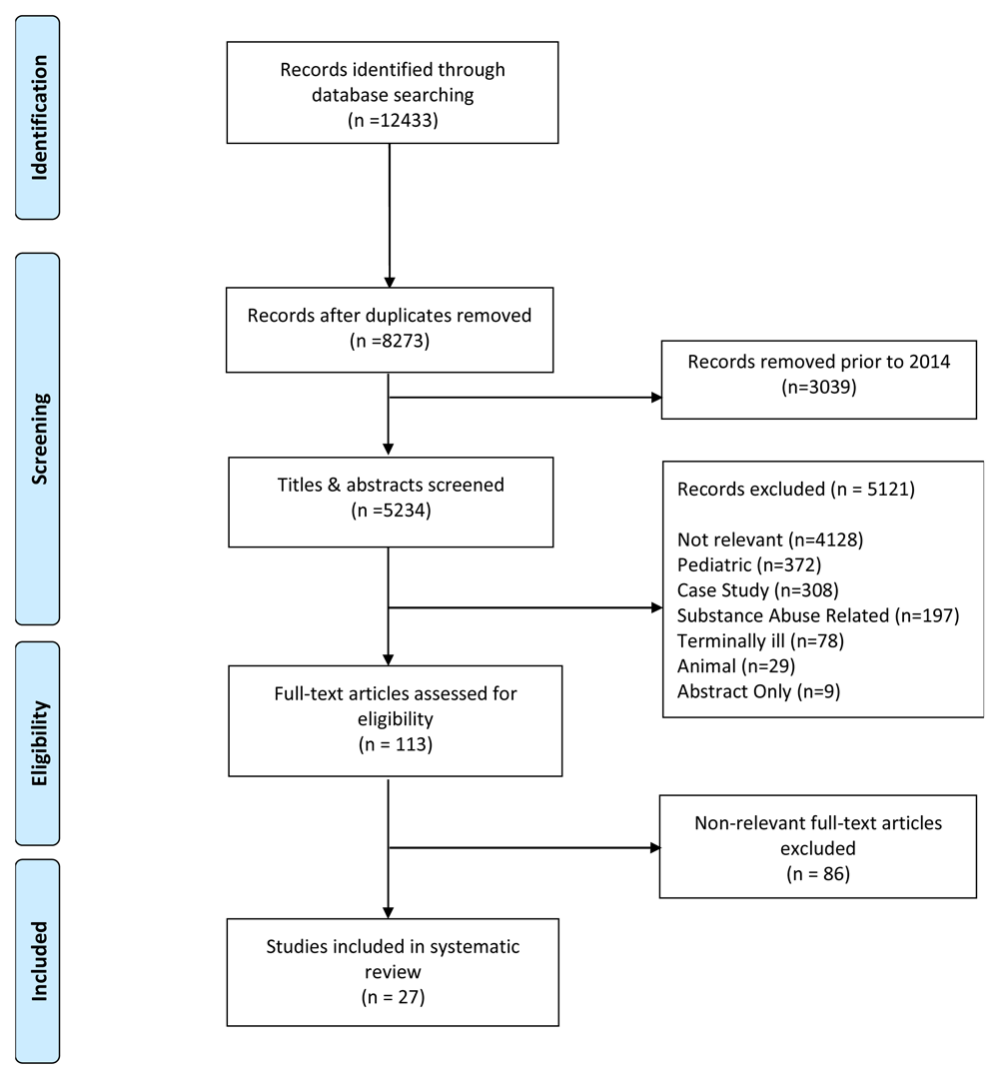
PRISMA Record Screening Flow Chart.

In addition to our search terms, search results were restricted to articles published in English within five years of the date of the search (Jan 1, 2014 to May 15, 2019). After retrieving articles within a ten year window as outlined in Extended Data ^20^, the authors decided to further reduce the range to within five years. This date range was chosen in order to provide a review of the most recent developments in this field of research. After search results were compiled and duplicates were removed, a total of 5234 articles were selected for title and abstract review. Four authors (JSC, HH, MMR, RB) screened the titles and abstracts and retrieved articles for eligibility, resulting in 113 articles selected for full text review. Three authors (JSC, HH, JL) then independently reviewed the full text of eligible articles, completed data extraction worksheets adapted from the Cochrane Review Group’s Data Extraction Form ( https://dplp.cochrane.org/data-extraction-forms), and assessed the articles for risk of bias using the Cochrane risk of bias tool ^21^. Elements of the data extraction worksheet included study design and setting, participant characteristics, details of the intervention and control groups, diagnostic tools, and patient outcomes ( *Extended data*, Supplementary Table 1) ^20^. Any disagreements were resolved by thoroughly discussing any points of concern. During the full text review 86 articles were removed because they failed to meet our inclusion criteria resulting in a total of 27 included articles ( Figure 1).

### Inclusion and exclusion criteria

Our review addresses non-pharmacological management strategies for delirium in the ICU. Included articles were those investigating non-pharmacologic interventions and their impact on delirium incidence, prevalence, duration, or severity in an adult (≥ 18 years) intensive care unit setting. Articles were excluded if they focused on non-human subjects, pediatrics, terminally ill subjects, withdrawal related delirium, case reports, or where no full-text article was available (abstract only). There were no restrictions on study design. Studies solely investigating delirium-free-coma-free days were excluded since it is not possible to review as a delirium-specific result. One multi-center study was excluded as both the frequency and method of assessment for delirium were not specified for all study centers, making it difficult to reliably compare the results with other trials ^22^. Another study was excluded because neither the screening process nor the cohort were described other than total number of patients enrolled, and there were no exclusion criteria noted to infer any characteristics of the selected population ^23^.

### Risk of bias assessment

In addition to data extraction using the Cochrane Review Group’s Data Extraction Form, a risk of bias assessment was performed by all authors on all included randomized controlled trials (RCTs) and randomized pilot studies. A risk of bias worksheet was developed by modifying Cochrane’s Risk of Bias Tool and articles were ranked as having a low, high, or unclear risk of bias ^21^. Disagreements were settled by discussion between the authors. A total of eleven included studies underwent this assessment. Details of the risk of bias assessment categories can be found in the *Extended data*, Supplementary Table 2 ^20^.

## Results

After searching the literature, 27 articles are included in our systematic review ^24– 50^ ( Figure 1). Study details of each reviewed trial are located in Table 1. The 27 included studies provide results on many distinct outcomes; however, only the delirium-related outcomes of incidence, prevalence, duration, and severity were reviewed ( Table 2– Table 5). Outcomes combining delirium and coma into the same statistic were excluded, as no delirium-specific results could be assessed outright. An overall summary of delirium outcomes can be found in Table 2.

**Table 1.** Study summary.

Study	Design	Population, n	Patient characteristics	Notable exclusion criteria	Intervention characteristics	Control characteristics	Method(s) of assessment & frequency	Pharmacologic aspect?
Álvarez *et al.* 2017	RCT, pilot	n=140	Non-intubated patients ≥60 years old, hospitalized within 24 hours in the ICU for postsurgical observation or for acute or chronic decompensated illness	Severe communication disorders; delirium before ICU admission; cognitive decline (defined with score >3.3 in the IQCODE and >6 points in the Spanish version of FAQ)	Early and intensive OT Details: two 40 minute sessions per day (one in the morning and one in the evening) for 5 consecutive days Components: polysensory stimulation; body positioning; cognitive stimulation; BADLs; stimulation of upper extremities; family engagement	Standard non- pharmacological prevention Components: reorientation twice daily; early mobilization 3 times daily; sight and hearing aids; environmental management (minimize physical restraints; clock and calendar); sleep protocols; caution with use of medications with the potential to cause delirium	CAM: duration & incidence of delirium (twice daily for 5 consecutive days) DRS: severity of delirium	Yes: avoidance of nighttime medications, anticholinergic drugs, and benzodiazepines
Arbabi *et al.* 2018	quasi- experimental	n=148	Patients >18 years admitted to general ICU	History of cognitive disorders such as dementia; alcohol abuse; impaired consciousness; history of psychiatric drugs; must not interrupt the admission drug use	Multicomponent bundle Components: staff education; educational posters; environmental changes: clock, calendar, family visits, appropriate lighting, eye & ear aids, light alarms instead of audio alarms, staff-patient interaction, hydration, early mobility	Usual care, details not specified.	CAM-ICU (twice daily, once in morning and once in evening)	Yes: staff educated on pharmacologic delirium treatments
Balas *et al.* 2014	pre-post, prospective cohort study	n=296	Patients ≥19 years old admitted to medical or surgical critical care service	No LAR present to provide consent within 48 hours of ICU admission; those who failed the safety screening or did not receive clearance from their physician	ABCDE bundle Components: (A) sedation awakening trial; (B) spontaneous breathing trial; (C) interdisciplinary coordination; (D) delirium monitoring & management; (E) early mobility	Usual care, details not specified.	CAM-ICU (every 8 hours)	None.
Bounds *et al.* 2016	retrospective study	n=159	Patients ≥18 years old and have stayed in the ICU >24 hours	Intracranial pressure increased >50% from first ICU measure for hospitalization; quadriplegia; GCS score < 8 without use of sedatives; comfort measures only as documented in the medical record as a medical order for life- sustaining treatment and/or palliative care; cardiopulmonary arrest resulting in death.	ABCDE bundle Components: (A) sedation awakening trial; (B) spontaneous breathing trial; (C) choice of medication & interdisciplinary coordination; (D) delirium prevention & management; (E) early mobility	Usual care, details not specified.	ICDSC (twice daily at 05:00 and 17:00)	Yes: medication management
Bryczkowski *et al.* 2014	pre-post, prospective intervention cohort study	n=123	All patients >50 years old consecutively admitted to the SICU for ≥24 h	Diagnosed with moderate to severe TBI, defined as a head AIS score of ≥3; transfer from jail or in police custody; history of dementia; patients whose delirium statuses were recorded as “unobtainable” or undocumented during their time in the SICU	Multicomponent bundle Components: protocol to lighten sedation; limit medications associated with delirium; sleep protocol: limit unnecessary PM care, lower lights and volume, massage, music, quiet time therapy; staff- patient-family education program	Usual care, details not specified.	CAM-ICU (at least every 12 hours, additional measurements after changes to routine, i.e. after a visit to radiology)	Yes: medication management
Campbell 2014	evidence-based project	n=58	Patients ≥18 years old, admitted to or in the ICU and placed on mechanical ventilation for at least 48 hours	Primary diagnosis of stroke with coma, or myocardial infarction with coma; pregnant; history of developmental disability or dementia; receiving therapeutic hypothermia; those dependent in activities of daily living prior to admission; actively dying	Early mobility protocol Details: introduction of progressive mobility exercises following successful interruption of sedation and awakening	Usual care, details not specified.	CAM-ICU (once daily at 08:00)	None.
Chai 2017	pre-post, quasi- experimental QI project	n=301	Any patient >18 years old admitted to a comprehensive mixed-ICU (medical, surgical, cardiac, neurology)	No exclusion criteria.	ABCDEF bundle Components: (A) assessment/ management of pain; (B) spontaneous awakening & breathing trials; (C) choice of sedation; (D) delirium monitoring & management; (E) early mobility; (F) family engagement	Details not specified.	CAM-ICU (at least once per shift)	Yes: pain management and choice of sedation
Damshens *et al.* 2018	RCT	n=80	Any patient >15 years old admitted to ICU trauma service	History of cognitive impairment, depression, taking psychotropic drugs, drug abuse, and alcohol abuse; patients with visual or hearing loss; ICU admission for <48 hours	Music therapy Details: two 45 minute sessions per day (once in morning and once at night); music tracks were mainly instrumental and selected by a music expert	Details not specified.	CAM-ICU (at least once per shift)	None.
Eghbali-Babadi *et al.* 2017	RCT	n=68	Non-intubated ICU patients 18–70 years old after elective open heart surgery, with immediate family available for intervention	History of addiction to drugs, alcohol, and cigarette smoking; presence of delirium, consciousness level disorder, or mental disease before surgery; history of blindness or deafness; family history for surgery *; death; post-surgery acute complications	Family visitation Details: additional 30–40 minute morning visitation session by an approved, study-educated immediate family member Components: visual and hearing aids; hand holding; reorientation; presence of meaningful personal items; encouragement to express feelings and regain independence	Visitation by hospital regulations, and visits were recorded.	CAM-ICU (twice daily, once in evening and once in morning after family visit)	None.
Fallahpoor *et al.* (2016)	action research study	n=100	Patients >18 years old admitted to ICU after elective CABG surgery	Blindness or deafness; history of mental illness, CVA, or kidney failure; return to operating room; bleeding or tamponade; requiring balloon pump or mitral valve repair; death	3-part management program: Before surgery: effective patient communication; identify and control delirium risk factors; staff and patient education; During surgery: identify delirium risk factors; stabilize hemodynamics; time management; After surgery: identify delirium risk factors; optimize environment; reorientation; effective communication; psychological & medical support; physical stability & activity; safety; sleep and sensory experiences	Details not specified.	CAM-ICU (every 8 hours)	Not specified.
Giraud *et al.* 2016	RCT, pilot time- cluster	n=223	ICU patients ≥70 years old after elective or urgent cardiac surgery	Severe visual impairment preventing self- recognition in a mirror; physical or communication barriers impeding intervention; severe mental disability; history of psychiatric illness requiring prior hospitalization	Structured mirror usage Details: use of and education on using mirrors to support mental status, attention, physical mobilization, and multisensory feedback and integration; use on awakening, change in mental status, and during nursing procedures and activity therapies Mirror details: (a) 23x41 cm personal mirror for viewing of the face; (b) 160x50 cm posture mirror to provide visual, proprioceptive feedback	Current standard post-surgical ICU care, including no prescriptions around the use of mirrors, and allowing control patients who brought a mirror from home to use it per their normal habits.	CAM-ICU (twice daily)	None.
Guo *et al.* 2016	RCT	n=160	Oral cancer patients aged 65–80 years scheduled for tumor resection surgery	History of CNS disorders or mental illness; MMSE score <24 or dementia of various etiologies; history of endocrine and metabolic disorders; recent use of glucocorticoids; alcohol or drug dependence; secondary surgery or severe infectious complications; illiteracy or language barriers; severe hearing or visual impairment; projected SICU stay ≤ 48 hours	Multicomponent non-pharmacologic intervention: Components: cognitive prehabilitation; post- operative reorientation; eye & ear aids; additional communication avenues for verbally impaired patients; decreased environmental noise; improved sleep protocols; eye masks & earplugs; limited restraint & catheter use; music therapy; immediate nasal feeding if able	Usual care consisted of standard hospital services provided by physicians, nurses, and support staff (e.g., physical therapists, pharmacists, and dietitians).	CAM-ICU (twice daily, once in morning and once in evening)	None.
Hamzehpour *et al.* 2017	RCT	n=100	ICU patients >18 years old	GCS ≤ 7; history of mental illness; death; ICU stay <24 hours; sedative injection	Roy adaptation model Components: delirium education; fluid and electrolyte balance; nutrition protocol; sleep protocol; activity and mobility protocol; monitoring of excretion, oxygen, circulatory, and endocrine condition	Routine care: performing physician orders; daily assessment of consciousness; systematic patient evaluation	NEECHAM confusion scale (twice daily, once in morning and once in evening)	Not specified.
Karadas & Ozdemir 2016	RCT	n=94	Patients ≥65 years old with an ICU stay of at least 24 hours	Presence of delirium before the procedure; amputated extremity; invasive mechanical ventilation; procedures limiting mobility; RASS score of -4 and -5; advanced osteoporosis; terminal illness; known cognitive disorders (dementia and psychosis); increased intracranial pressure; active gastrointestinal system bleeding; arrhythmia; active myocardial ischemia	ROM exercises Details: 10 repetitions per exercise for approximately 30 min once daily; if patient did not tolerate the intervention, it ceased & continued the next day Components: passive, assisted-active, or active ROM exercises performed on the four extremities in the supine position	Usual care, details not specified.	CAM-ICU (once daily)	None.
Khan *et al.* (2014)	pre-post, implementation study	n=702	Mechanically ventilated ICU patients ≥ 18 years old	Hearing impaired; legally blind; admitted with alcohol intoxication; prisoners; diagnosed as having an Axis 1 Psychiatric disorder	‘Wake Up and Breathe' protocol Components: daily sedation interruption & spontaneous awakening trial, followed by spontaneous breathing trial depending on the patient’s response to the former	Continuous analgesic and sedative infusions based on physicians' discretion.	CAM-ICU (twice daily)	Yes: sedation management
Kram *et al.* (2015)	pre-post, implementation study	n=83	All adult patients admitted to the ICU	Safety screening failures; patients without clearance from their attending surgeon; physicians could write an order out of any bundle component they believed was not in the patient’s best interest	ABCDE bundle Components: (A & B) spontaneous awakening and breathing trials; (C) interdisciplinary coordination; (D) delirium screening & management; (E) early mobility	Details not specified.	ICDSC (twice daily at 05:00 and 17:00)	Yes: sedation management
Lisann- Goldman *et al.* 2017	mixed-methods pilot study	n=25	Patients ≥40 years old, with planned ICU admission after elective cardiac surgery with cardiopulmonary bypass, and a MMSE score of ≥24	Intracranial or emergency surgery; severe visual or auditory disorder or handicaps; illiteracy or minimal reading or writing ability; presence of a major, uncontrolled psychiatric condition; severe cognitive impairment	Mindfulness exercises Pre-operative: discussion exercises with the study PI; listen to audio discussing Langerian mindfulness (8 minutes 48 seconds) Post-operative: discussion exercises with the study PI, twice daily in the morning and afternoon	Preoperative: Engaged in discussion related to cardiac surgery and then listened to audio describing the step-by- step process of undergoing cardiac surgery (8 minutes 34 seconds) Post-operative: neutral conversation on the same twice daily schedule	CAM-ICU (twice daily)	None.
Mailhot *et al.* 2017	randomized pilot study	n=30	All patients admitted to the surgery ICU or surgery unit with: (1) post- surgical delirium confirmed by a DSM-V medical diagnosis; (2) undergoing either CABG or heart valve surgery; and (3) has a family caregiver available for scheduled bedside visits	Planned transfer to another hospital less than 3 days after delirium onset; preoperative diagnosis of cognitive impairment; irreversible postoperative cognitive damage	Family caregiver Details: an approved, study-educated family member applied bedside strategies to aid the patient in reorientation, observe and communicate signs of delirium with nursing staff, and present family memories	Both groups received usual care, including pharmacological and non- pharmacological interventions suggested in best practice guidelines described in the Registered Nurses’ Association of Ontario guidelines (RNAO, 2016).	CAM-ICU (once daily) DI (once positive delirium result is recorded) DSM-V (confirm delirium diagnosis once positive ICDSC result is recorded) ICDSC (once daily)	Yes: use of pharmacological interventions in usual care; usual care is applied to both study groups.
Moon & Lee 2015	RCT, single-blind	n=123	Patients ≥18 years old on day of ICU admission with ICU stay of ≥48 hours	Persistent RASS score of -4 or -5; severe visual and auditory problems; serious psychiatric or neurologic diagnosis; score of ≤23 on the MMSE-Korean; admission to the isolation ward due to infection; death or discharge on day of admission; inability to conduct CAM-ICU when in a very violent status & RASS +3 or +4	Multicomponent bundle Components: cognitive assessment & reorientation; sensory aids; indirect night lighting; consistent care staff; items from home; minimize bed relocation; address & manage delirium risk factors early; nutritional, fluid, electrolyte balance; early ambulation; increased awareness of drugs associated with delirious side effects; early infection detection; limit catheter use; monitor for hypoxemia; pain control	Typical nursing care included regular checking of consciousness and orientation without attempting to (1) reorient the patient; (2) communicate using nonverbal communication skills; (3) provide visual or hearing aids; (4) assign the same nurse in charge throughout stay; (5) minimize bed movement; or (6) carefully use particular medications (e.g., anticholinergic agents and opiates)	CAM-ICU (frequency not specified)	Yes: careful use of drugs associated with delirious side effects; pain control
Munro *et al.* 2017	RCT, prospective	n=30	Patients >18 years old within 24 hours of ICU admission	Anticipation of imminent patient death; medical contraindication to the intervention (i.e. psychiatric auditory hallucinations, or profound deafness); inability to speak either English or Spanish	Automated reorientation messages Details: reorientation script recorded by either a family member or a bilingual female unknown to the subject; played 8x from 09:00-16:00 stating the subject’s preferred name & that the message is recorded; the following details were randomly ordered to prevent repetition: information about ICU environment, expected visual and auditory stimuli, availability of staff & family	Usual care, details not specified.	CAM-ICU (twice daily)	None.
Parry *et al.* 2014	case-matched control study	n=16	Patients ≥18 years old admitted to the ICU with a diagnosis of sepsis, mechanically- ventilated for >48 hours, and in ICU for ≥4 days	Physical limitations (presence of external fixator, pacemaker, defibrillator, open wound or skin abrasions); obesity (BMI>40, too heavy for cycling machine); anticipation of imminent patient death	Functional electrical stimulation (FES) cycling Details: supine, motorized cycle ergometer attached to a current-controlled stimulator(20-60 min once daily 5x a week); stimulation occurred at specific times, based on normal activation patterns regulated by the bicycle software & causing visible muscle contraction (quadriceps, hamstrings, gluteals, calves)	Usual care following institutional protocols for resuscitation & sepsis management, routine physiotherapy once subject was considered awake using De Jonghe 5-point criteria	CAM-ICU (once daily)	None.
Pun *et al.* 2019	prospective, multicenter cohort study via national QI collaborative	n=10840	Any adult patient admitted to medical, surgical, cardiac, or neurologic ICU for at least 2 consecutive 24- hour days	Death or ICU discharge within 24 hours of ICU admission, active life support withdrawal and/or comfort-care measures within 24 hours of ICU admission	ABCDEF bundle Components: (A) assess/prevent/manage pain; (B) spontaneous awakening & breathing trials; (C) choice of analgesia/sedation; (D) delirium monitoring & management; (E) early mobility; (F) family engagement	NA: analytic comparison is between complete bundle performance and partial bundle performance	CAM-ICU or ICDSC (≥2 times daily)	Yes: pain management and choice of analgesia/ sedation
Rivosecchi *et al.* 2016	pre-post, prospective observational QI project	n=483	Any patient admitted to the medical ICU	Any amount of time admitted to an ICU (internal or external) before MICU admission; documented history of cognitive impairment; already admitted to MICU when study period began; MICU stay ≤24 hours; presented to the MICU delirious; no recorded ICDSC measurements	M.O.R.E. bundle + staff education Components: (M) music; (O) opening/closing of blinds; (R) reorientation/ cognitive stimulation; (E) eye/ear care	Daily bedside multidisciplinary rounds, sedation algorithms, mobilization protocols, and every 4-hour delirium screening using the ICDSC.	ICDSC (every 4 hours)	Yes: sedation algorithms
Rosa *et al.* 2017	pre-post study, prospective	n=286	any patient ≥18 years old admitted to the ICU	Patients with aphasia, pre-existing delirium, exclusively palliative treatment at ICU admission, expected ICU stay <24 hours, those who remained unarousable ≥48 hours (RASS –4 or –5), and those readmitted to the ICU after enrollment in the study.	Extended visitation hours Details: two or fewer visitors at a time, for 12 hours/day (09:00- 21:00); families allowed to participate in multidisciplinary bedside rounds; no time limit for terminal patients, conflicts, or delirium	Regular visitation schedule Details: two or fewer family visitors at a time for 4.5 hours per day, broken up into three visitation blocks (09:00-11:00, 16:00-17:30, 21:00-22:00); no time limit for terminal patients, conflicts, or delirium	CAM-ICU (twice daily, once every 12 hours)	None.
Simons *et al.* 2016	RCT	n=734	any patient ≥18 years old admitted to the ICU	An expected ICU stay of <24 hours; anticipated life expectancy <48 hours; severe hearing or visual impairment; severe mental impairment	Dynamic Lighting Application (DLA) Details: gradual exposure to blueish-white light from 07:00-11:30 and 13:30- 16:00 (peak 1700 lux, 4300 K); from 11:30-13:30 the light was dimmed to 300 lux, 3000 K.	The control group was consistently exposed to 300 lux, 3000 K lighting.	CAM-ICU (at least 3 times daily) non-ICU patients: DOS scale, consultation with geriatrician, treatment with pharmacological agents for suspected delirium (frequency not specified)	None.
Sullinger *et al.* 2017	pre-post study, retrospective, observational	n=89	critically ill patients ≥18 years old with acute delirium in an open surgical- trauma ICU	Missing or incomplete CAM- ICU assessments; pregnancy; history of dementia, schizophrenia, bipolar disease or a prolonged QTc at baseline (>500 ms regardless of gender).	Management bundle + nursing education Components: non-pharm interventions (replacing vision/hearing aids, healing arts consult [massage therapy, craniosacral therapy, therapeutic touch, music therapy], mobility plan [repositioning, sitting, ambulation], light and blinds adjustment, revised quiet hours [13:00- 15:00 and 22:00- 04:00], earplugs, family presence); initiate anti- psychotic medications if non-pharmacologic therapies fail	Details not specified.	CAM-ICU (twice daily, once per 12- hour shift)	Yes: initiate anti-psychotic medications if non- pharmacologic therapies fail
Zhang *et al.* 2017	pre-post study, prospective	n=278	any patient ≥18 years old admitted to the cardiothoracic ICU after CABG	ICU stay of ≤24 hours; history of mental disease or delirium at admission; patient could not awaken from surgery within the first 24 hours after surgery	Management bundle Components: delirium risk factor screening & modification (pain control, early catheter removal, reorientation, increased family visits, minimizing care-related interruptions, comfortable nursing, monitoring for sleeping difficulties)	Details not specified.	CAM-ICU: delirium (not specified) DSR-R-98: severity (when a positive CAM-ICU is recorded)	Yes: medication management

***Abbreviations:*** *BADLs: basic activities of daily living; CABG: coronary artery bypass graft; CAM: Confusion Assessment Method; CNS: central nervous system; CVA: cerebrovascular accident; DI: Delirium Index; DOS scale: Delirium Observation Screening scale; DRS/DRS-R-98: Delirium Rating Scale-Revised-98; DSM-V: Diagnostic and Statistical Manual of Mental Disorders, 5th Edition; FAQ: Functional Activity Questionnaire; GCS: Glasgow Coma Score; ICDSC: Intensive Care Delirium Screening Checklist; ICU: intensive care unit; IQCODE: Informant Questionnaire on Cognitive Decline in the Elderly; LOS: length of stay; MMSE: Mini-Mental State Examination; OT: occupational therapy; QI: quality improvement; RASS: Richmond Agitation-Sedation Scale; RCT: randomized controlled trial; SICU: surgical ICU.* * exclusion criteria of 'family history for surgery': cannot confirm whether for elective open heart surgery, or surgery in general.

**Table 2.** Summary of delirium outcomes.

Individual interventions	Incidence	Prevalence	Duration	Severity
automated reorientation ^44^	--	--	yes*	--
dynamic lighting ^48^	n.s.	--	n.s.	--
family, caregiver ^42^	NA	--	NA	n.s.
family, RVM vs EVM ^47^	yes	--	--	--
family, additional structured visit ^32^	yes	--	--	--
mindfulness exercises ^41^	NA	--	--	--
mirrors ^34^	n.s.	--	n.s.	--
mobility, early and intensive OT ^24^	yes	--	yes	n.s.
mobility, early ^29^	n.s.	--	n.s.	--
mobility, ROM exercises ^38^	n.s.	--	n.s.	--
mobility, FES ^45^	n.s.	--	yes	--
music therapy ^31^	n.s.	--	--	--
Bundled interventions	Incidence	Prevalence	Duration	Severity
ABCDE ^26^	--	yes	yes*	--
ABCDE ^27^	--	yes	yes	--
ABCDE ^40^	--	NA	--	--
ABCDEF ^30^	yes	--	--	--
ABCDEF ^37^	yes	--	--	--
M.O.R.E. ^46^	yes	--	yes	--
multicomponent ^25^	yes	--	yes	--
multicomponent ^28^	n.s.	--	yes	--
multicomponent ^43^	n.s.	--	--	--
multicomponent ^49^	--	--	yes	--
multicomponent non-pharmacologic ^35^	yes*	--	yes	--
post-CABG delirium management ^33^	yes	--	--	--
risk factor screening & target modification ^50^	yes	--	n.s.	n.s.
Roy adaptation model ^36^	yes*	--	--	yes*
'Wake Up and Breathe' protocol ^39^	n.s.	n.s.	--	--

**Legend:** n.s., not significant, p>0.05; yes, significant, p<0.05; NA, not analyzed for significance; --, not measured in this study; yes*, some measured time-points are significant.

**Table 3.** Results on incidence and prevalence of delirium.

Incidence					
Intervention	Number of patients enrolled	Group Results			Statistical Results
ABCDEF bundle ^30^ ^b^	n=351 (CG n=151; IG n=150)	**Day** **08:00–11:00**	CG 77 (51%) IG 16 (10.7%)		χ2(1) = 57.32 p <.001 *
		**Night** **20:00–23:00**	CG 89 (58.9%) IG 27 (18%)		χ2(1) = 53.25 p <.001 *
ABCDEF bundle, complete vs partial performance ^37^ ^i^	n=10840		Not reported		AOR 0.60 (0.49–0.72) p < 0.0001
Dynamic lighting ^48^ ^c^	n=734 (CG n=373; IG n=361)		CG 123 (33%) IG 137 (38%)		OR 1.24 (0.92, 1.68) † p=0.16
Family, additional structured visit ^32^ ^c^	n=68 (CG n=34; IG n=34)	**Day 2** **10:00**	CG 8 (23.53%) IG 4 (11.76%)		χ2=3.98 p=0.04 *
		**Day 2** **17:00**	CG 11 (32.35%) IG 4 (11.76%)		χ2=8.38 p<0.05 *
		**Day 3** **10:00**	CG 7 (20.58%) IG 3 (8.83%)		χ2=4.12 p=0.03 *
Family, caregiver ^42^ ^a^	n=30 (CG n=14; IG n=16)		CG 71.4% IG 43.8%		NA
Family, restricted visitation vs extended visitation ^47^ ^c^	n=286 (CG n=141; IG n=145)		CG 29 (20.5%) IG 14 (9.6%)		RR 0.50 (0.26, 0.95) † p=0.03 *
Mindfulness exercises ^41^ ^d^	n=25 (CG n=13; IG n=12)		CG 0 (0%) IG 0 (0%)		NA
Mirrors ^34^ ^c^	n=223 (CG n=108; IG n=115)		CG 17 (16%) IG 20 (17%)		OR 1.15 (0.54,2 .43) † p=0.705
Mobility, early & intensive OT ^24^ ^c^	n=140 (CG n=70; IG n=70)		CG 14 (20%) IG 2 (3%)		p=0.001 *
Mobility, early mobility ^29^ ^c^	n=58 (CG n=27; IG n=31)		CG 24 (89%) IG 29 (93.5%)		χ2(1, N=38) = 0.398 p=0.53
Mobility, FES cycling therapy ^45^ ^c^	n=16 (CG n=8; IG n=8)		CG 7 (87%) IG 2 (25%)		p>0.05
Mobility, ROM exercises ^38^ ^c^	n=94 (CG n=47; IG n=47)		CG 10 (21.3%) IG 4 (8.5%)		χ2=3.02 p>0.05
M.O.R.E. bundle + nursing education ^46^ ^e^	n=483 (CG n=230; IG n=253)		CG 36 (15.7%) IG 24 (9.4%)		p=0.04 *
Multicomponent bundle (staff education & environmental changes) ^25^ ^c^	n=148 (CG n=69; IG n=79)		CG 50 (72.46%) IG 30 (37.97%)		p=0.01 *
Multicomponent bundle + education ^28^ ^c^	n=123 (CG n=57; IG n=66)		CG 27 (47%) IG 38 (58%)		p=0.26
Multicomponent bundle ^43^ ^c^	n=121 (CG n=63; IG n=60)		CG 21 (33.3%) IG 12 (20.0%)		p=0.10
Multicomponent, non- pharmacologic bundle ^35^ ^c^	n=160 (CG n=79; IG n=81)	**Day 1**	CG 13 (16.25%) IG 4 (7.50%)		p=0.035 *
		**Day 2**	CG 9 (11.25%) IG 5 (6.25%)		p=0.374
		**Day 3**	CG 4 (5.00%) IG 1 (1.25%)		p=0.364
		**Total**	CG 25 (31.25%) IG 10 (15.00%)		p=0.006 *
Music therapy ^31^ ^c^	n= 80 (CG n=40; IG n= 40)		CG 16 (40%) IG 15 (37.5%)		χ2=0.053 p>0.818
Post-CABG delirium management bundle ^33^ ^b,c^	n=100 (CG n=50; IG n=50)	**Overall** **incidence**	CG 34 (68%) IG 19 (38%)		p=0.001 * -30% change
		**Patients** **with** **‘x’ number** **of** **delirious** **events**	**0**	CG 16 (32%) IG 31 (62%)	
			**1–3**	CG 24 (48%) IG 12 (24%)	
			**4–7**	CG 8 (16%) IG 5 (10%)	
			**8+**	CG 2 (4%) IG 2 (4%)	
			**Overall**	NA	*p=0.008*
Risk factor screening & target modification ^50^ ^c^	n=278 (CG n=137; IG n=141)		CG 41 (29.93% IG 19 (13.48%)		χ2=11.112 p=0.001 *
Roy adaptation model ^36^ ^f^	n=100 (CG n=50; IG n=50)	**Days 1–6** **(AM & PM)**	See full reference text.		p>0.05
		**Day 7 AM**	CG 31 (68.9%) ‡ IG 15 (36.6%) ‡		p<0.008 *
		**Day 7 PM**	CG 30 (61.9%) ‡ IG 20 (42.9%) ‡		p<0.05 *
'Wake Up and Breathe' protocol ^39^ ^g^	n=702 (CG n=262; IG n=440)		CG 14 (23.0%) IG 33 (19.6%)		AOR 0.718 (0.326,1.578) † p=0.40
Prevalence					
Intervention	Number of patients enrolled	Group Results		Statistical Results	
ABCDE bundle ^26^ ^c^	n=296 (CG n=146; IG n=150)		CG 91 (62.3%) IG 73 (48.7%)	p=0.03 *	
ABCDE bundle ^27^ ^e^	n=159 (CG n=80; IG n=79)		CG 38% IG 23%	p=0.01 *	
ABCDE bundle ^40^ ^e^	n=83 (CG n=47; IG n=36)		CG NA IG 7 (19%)	NA	
‘Wake Up and Breathe’ protocol ^39^ ^h^	n=702 (CG n=262; IG n=440)		CG 94 (66.7%) IG 167 (55.3%)	AOR 0.650 (0.413, 1.022) † p=0.06	

**Abbreviations:** AOR: adjusted odds ratio; CABG: coronary artery bypass graft; CG: control group; FES: functional electrical stimulation; IG: intervention group; FES: functional electrical stimulation; NA: not analyzed; OR: odds ratio; OT: occupational therapy; ROM: range of motion; RR: relative risk. **Legend:** a = Percent of patients with at least one positive CAM-ICU screening; b = Number of recorded delirium events; c = Number of patients with at least one positive CAM-ICU screening; d = Number of patients with at least one positive CAM-ICU screening, patients with RASS -4 or -5 counted as ‘not delirious’; e = Number or percent of patients with at least one positive ICDSC screening; f = Number of patients with at least one NEECHAM score of < 25; g = Number of patients with a positive CAM-ICU after an initial negative result; h = Number of patients with any CAM-ICU positive result; i = not reported; * = significant difference, p<0.05; † = 95% confidence interval; ‡ = contradictory numbers reported, see referenced text.

**Table 4.** Results on duration of delirium.

Duration				
Intervention	Number of patients enrolled	Duration measurement	Group Results	Statistical Results
ABCDE bundle ^26^	n=296 (CG n=146; IG n=150)	duration in days median (IQR)	CG 3 (1, 6) IG 2 (1, 4)	p=0.52
		% ICU days spent delirious median (IQR)	CG 50% (30, 64.3) IG 33.3% (18.8, 50)	p=0.003 ^*^
ABCDE bundle ^27^		number of days a patient had positive ICDSC score mean ± SD (range)	CG 3.8 ± 2.9 (1.0, 14.0) IG 1.72 ± 0.8 (1.0, 4.0)	p<0.001 ^*^
Automated reorientation ^44^	n=30 (CG n=10; UG n=10; FG n=10)	delirium-free days mean (SD)	CG 1.6 (1.13) UG 1.6 (1.07) FG 1.9 (0.99)	p=0.0437 ^*^
		days of delirium mean (SD)	CG 0.9 (1.28) UG 0.6 (0.84) FG 0.3 (0.48)	p>0.05
Dynamic lighting ^48^	n=734 (CG n=373; IG n=361)	duration in hours median (IQR)	CG 2 (1, 5) IG 2 (2, 5)	p=0.87
Family, caregiver ^42^	n=30 (CG n=14; IG n=16)	duration in days mean (SD)	CG 4.14 (4.04) IG 1.94 (1.34)	--
Mirrors ^34^	n=223 (CG n=108; IG n=115)	duration in days median (IQR [range])	CG 2 (1, 8 [1, 3]) IG 1 (1, 3 [1, 25])	RR 0.66 (0.26, 1.70) † p=0.393
		proportion of ICU stay mean (SD)	CG 0.65 (0.29) IG 0.54 (0.30)	Co-eff -0.10 (-0.67, 0.47) † p=0.729
Mobility, early & intensive OT ^24^	n=140 (CG n=70; IG n=70)	ratio of delirium duration to exposure time	CG IRR 6.66 (5.23, 8.3) † IG IRR 0.15 (0.12, 0.19) †	CG p =0.000 ^*^ IG p=0.000 ^*^
Mobility, early ^29^	n=58 (CG n=27; IG n=31)	duration in days mean ± SD (range)	CG 2.70 ± 2.18 (0, 9) IG 3.58 ± 2.68 (0, 9)	p=0.18
Mobility, FES ^45^	n=16 (CG n=8; IG n=8)	duration in days median (IQR)	CG 6.0 (3.3, 13.3) IG 0.0 (0.0, 3.0)	p=0.042 ^*^
Mobility, ROM exercises ^38^	n=94 (CG n=47; IG n=47)	duration in hours median (range)	CG 38 (9, 120) IG 15 (3, 144)	Z= -0.997 p>0.05
M.O.R.E. bundle ^46^	n=483 (CG n=230; IG n=253)	duration in hours median (IQR)	CG 20 (9.5, 37) 16.1% of ICU LOS IG 16 (8, 24) 9.6% of ICU LOS	p<0.001 ^*^
Multicomponent bundle (staff education & environmental changes) ^25^	n=148 (CG n=69; IG n=79)	% of days with delirium mean ± SD	CG 35.84 ± 39.31 IG 26.18 ± 35.38	p=0.001 ^*^
Multicomponent bundle + education ^28^	n=123 (CG n=57; IG n=66)	delirium-free days out of 30 mean (range)	CG 24 (22, 26) † IG 27 (25, 28) †	p=0.002 ^*^
Multicomponent non- pharmacologic bundle ^35^	n=160 (CG n=79; IG n=81)	duration in hours mean ± SD	CG 60.2 ± 15.8 IG 28.1 ± 8.6	p<0.001 ^*^
Multicomponent bundle + nursing education ^49^	n=89 (CG n=38; IG n=51)	number of delirious days mean ± SD	CG 8.2 ± 5.7 IG 4.5 ± 4.4	p<0.001 ^*^
Risk factor screening & target modification ^50^	n=278 (CG n=137; IG n=141)	duration in days, from first positive CAM-ICU to recovery (2 consecutive days with negative CAM-ICU)	all durations (1–5 days), both groups	p=0.876

***Abbreviations:*** *CG: control group; FES: functional electrical stimulation; FG: family voice group; IG: intervention group; IQR: interquartile range; IRR: incidence risk ratio; OT: occupational therapy; ROM: range of motion; RR: relative risk; SD: standard deviation; UG: unknown voice group.* **Legend:** * = significant, p<0.05; † = 95% confidence interval.

**Table 5.** Results on severity of delirium.

Severity					
Intervention	Number of patients enrolled	Measurement	Group Results		Statistical Results
Family, caregiver ^42^	n=30 (CG n=14; IG n=16)	DI mean (SD)	Overall (Days 1–3)	NA	p=0.27
			Day 1	CG 2.07 (4.05) IG 10.56 (3.5)	NA
			Day 2	CG 8 (6.34) IG 5.38 (5.45)	NA
			Day 3	CG 5.5 (7) IG 3.43 (4.96)	NA
Mobility, early & intensive OT ^24^	n=140 (CG n=70; IG n=70)	DRS mean (range)		CG 10 (8, 13) † IG 9 (6, 12) †	p=0.7
Risk factor screening & target modification ^50^	n=278 (CG n=137; IG n=141)	DRS-R-98 Number (%)	Overall (Results from all 3 severity groups)	NA	Z= -0.792 p=0.428
			Mild	CG 10 (7.30) IG 7 (4.96)	NA
			Moderate	CG 21 (15.33) IG 8 (5.67)	NA
			Severe	CG 10 (7.30) IG 4 (2.84)	NA
Roy adaptation model ^36^	n=100 (CG n=50; IG n=50)	NEECHAM Confusion Scale	Day 1–3, AM & PM (6 time points)	See full reference text.	p>0.05
			Day 4–7, AM & PM (8 time points)	See full reference text.	p≤0.028 * (range p=0.000 to p=0.028)

***Abbreviations:** DI: Delirium Index; DRS: Delirium Rating Scale; DRS-R-98: Delirium Rating Scale-Revised-98; NA: not analyzed; OT: occupational therapy.* **Legend:** * = significant difference, p<0.05; † = confidence interval not specified.

Of these 27 articles, 24 assessed incidence and/or prevalence within their cohorts ^24– 43, 45– 48, 50^, 16 assessed for duration ^24– 28, 34, 35, 38, 42, 44– 46, 48– 50^, and four for severity ^24, 36, 42, 50^. Additionally, 12 focused on the effect of single interventions ^24, 29, 31, 32, 34, 38, 41, 42, 44, 45, 47, 48^ while 15 considered bundled, multicomponent interventions ^25– 28, 30, 33, 35– 37, 39, 40, 43, 46, 49, 50^. Individual interventions included mobility protocols, distinct family visiting policies, dynamic lighting, music therapy, automated reorientation messages, mindfulness exercises, and the structured use of mirrors in recovery. These individual interventions also comprised multiple components of the bundled interventions. A summary of study details can be found in Table 1.

Measurements for incidence, prevalence, and duration were based upon multiple methods of delirium screening, including the Confusion Assessment Method (CAM), CAM-ICU, Intensive Care Delirium Screening Checklist (ICDSC), and Neelon and Champagne (NEECHAM) scales. Incidence and prevalence were similarly defined in all studies except for one, and are recorded separately in Table 3; only one study looked at both incidence and prevalence ^39^. Severity was assessed by using the Delirium Index (DI), the Delirium Rating Scale (DRS), the Revised Delirium Rating Scale (DRS-R-98), and NEECHAM scale ( Table 5).

Of the 27 included studies, 11 were RCTs or randomized pilot studies ^24, 31, 32, 34– 36, 38, 42– 44, 48^, eight were pre-post prospective studies ^26, 28, 39, 40, 46, 47, 49, 50^, and two were quasi-experimental ^25, 30^. The remaining six were a case-matched control study, an evidence based protocol, a mixed-methods pilot study, a prospective multicenter cohort study, a retrospective cohort study, and an action research study ^27, 29, 33, 37, 41, 45^.

### Risk of bias assessment

The ten RCTs and the randomized pilot study underwent a risk of bias assessment performed by all authors. Risks of bias fell into five major groups (selection bias, performance bias, detection bias, attrition bias, and reporting bias), and based on a study’s scores in each of these groups it was labeled as having an overall high, low, or unclear risk of bias. Four were considered low risk of bias ^24, 34, 42, 48^, two had a high risk of bias ^32, 44^, and five had an unclear risk of bias ^31, 35, 36, 38, 43^. The most common source of bias was performance bias due to the impossibility of blinding participants or personnel to certain treatments. Common sources of unclear and high risk of bias included the methods of randomization and allocation concealment, as well as how missing data was handled.

### Individual interventions

***Early mobility***. The effect of early mobility protocols on delirium was the most commonly studied individual intervention. Four of the studies included in our review individually assessed the efficacy of early mobility ^24, 29, 38, 45^ in treating and preventing delirium; of these, two were RCTs ^24, 38^, one was an evidence-based project ^29^, and one was a case-matched control study ^45^. They assessed delirium through CAM ^24^ and CAM-ICU ^29, 38, 45^.

The pilot RCT performed by Álvarez *et al.* investigated the effect of early mobilization through early and intensive occupational therapy (OT), including polysensory stimulation, body positioning, cognitive stimulation exercises, basic activities of daily living, upper extremity motor exercises, and family involvement, on non-intubated, elderly patients (≥ 60) in addition to the study center’s standard, non-pharmacological delirium prevention care ^24^. Delirium associated outcomes included incidence, duration, and severity; they found significant differences in incidence and duration of delirium, with both p-values ≤ 0.001, but no significant difference in severity ( Table 3– Table 5).

Another RCT by Karadas and Ozdemir assessed the effect of range of motion (ROM) exercises on delirium in elderly ICU patients (≥ 65 years) ^38^. Interventional care included ROM exercises for 30 minutes daily after establishing the patient’s ability to complete 10 repetitions on each of the four extremities while lying in bed. They reported no statistically significant differences between cohorts for delirium associated outcomes ( Table 3 & Table 4).

Campbell addressed early mobilization in mechanically ventilated ICU patients with an evidence-based project ^29^. They measured the effect of a tiered protocol of ROM exercises, bed mobility exercises, seated balance activities, transfer activities (such as bed to chair), standing exercises, and ambulation on delirium incidence and duration but found neither to be significant ( Table 3 & Table 4).

The effectiveness of functional electrical stimulation (FES) to promote mobility and recovery in mechanically ventilated patients with sepsis was evaluated by Parry *et al.* in a case-matched control study ^45^. The intervention included use of a motorized cycle ergometer to directly stimulate four major lower limb muscles (quadriceps, hamstrings, gluteals, and calves) five times weekly for 20–60 minutes a session dependent on the individual patient’s tolerance. While delirium incidence was not significantly affected ( Table 3), the median days of delirium differed between arms (6.0 in control and 0.0 in intervention) ( Table 4).

***Family involvement***. Of the 12 studies in our review which focused on individual interventions, three studied the effect of family involvement on delirium in adult ICU patients. One was a randomized pilot study ^42^, one an RCT ^32^, and one was a pre-post study ^47^. All three studies utilized CAM-ICU in their assessment of delirium.

Mailhot *et al.* constructed a randomized pilot study to explore the effect of a family caregiver (FC) assisting with delirium management after being ‘mentored’ (MENTOR_D) by nurses in the ‘MENTOR_D’ intervention ^42^. They assessed the efficacy of this intervention on all delirious, adult coronary artery bypass graft (CABG) patients admitted to the surgical ICU by measuring the outcomes of duration, occurrence, and severity of delirium over three days ^42^. This intervention enrolled 14 patient-nurse (control) care dyads and 16 patient-FC care dyads, which had the FC apply bedside strategies to aid the patient in reorientation. In addition to reorientation, the FC was asked to observe and communicate signs of delirium with nursing staff, present family memories, and speak clearly and simply. Delirium duration and occurrence on post-operative Day 2 improved clinically between groups (duration, mean days from 4.14 to 1.94; occurrence, from 71.40% to 43.80%); however, this result was not assessed for statistical significance and the severity result was not found to be significant ( Table 3– Table 5).

The RCT performed by Eghbali-Babadi *et al.* investigated a modified family visitation policy, implementing an additional 30–40 minute special visit by an approved family member, and its effect on delirium incidence in non-intubated adults aged 18–70 after elective open heart surgery ^32^. They found a statistically significant reduction in delirium incidence in the intervention group with a p-values of 0.04, <0.05, and 0.03 at three different time points ( Table 3).

Rosa *et al.* also measured the effect of a modified family visitation policy on delirium incidence, although their population was less restrictive and included any adult ICU patient ^47^. Their pre-post study included the extension of visitation hours from 4.5 hours per day over three visitation blocks to 12 hours per day between 09:00–21:00. This resulted in a statistically significant difference in delirium incidence, improving from 20.5% to 9.6% ( Table 3).

***Environmental approaches (lighting, music therapy, automated reorientation)***. Three RCTs assessed the impact of environmental factors on delirium in the ICU, assessed by CAM-ICU, through manipulation of light ^48^, music therapy ^31^, or automated reorientation ^44^.

In Simons *et al.*’s dynamic lighting application RCT, adult ICU patients were exposed to variations in high intensity, blueish-white lighting while delirium incidence and duration were measured ^48^. The intervention group was exposed to a peak of 1700 lux (brightness)/4300 K (color temperature) from 09:00–11:30 and 13:30–16:00, and a daytime minimum of 300 lux/3000 K from 11:30–13:30; the control group was exposed solely to 300 lux/3000 K ( Table 1). Neither the cumulative incidence of ICU-acquired delirium nor the duration were significantly affected, and the trial was ended early after the intervention was deemed futile ( Table 3 & Table 4).

In another RCT, Damshens *et al.* introduced therapeutic music selected by a music expert, twice a day for 45 minutes to assess the effect on delirium incidence in adults admitted to ICU trauma service ^31^. Patients in the control group received conventional care for the duration of their admission. There was no resultant change to delirium incidence between the two groups ( Table 3).

Munro *et al.* developed a novel patient reorientation strategy in an RCT, which utilized bilingual (Spanish or English) messages pre-recorded by either family members or females unknown to the adult ICU subjects ^44^. The recordings included an introduction with the patient’s name and location, with several additional randomly ordered statements in order to reorient the patient to their unfamiliar surroundings and reason for hospitalization. All three arms (two intervention groups and one control group) were compared and it was found that the family voice group had a significant improvement in delirium free days (p= 0.0437) but not mean days of delirium ( Table 4).

***Self-involvement approaches (mirror usage, mindfulness exercises)***. The remaining two studies on the effect of individual interventions assessed the impact of self-involvement approaches, including mirror usage ^34^ and mindfulness exercises ^41^, on ICU delirium measured by CAM-ICU. One study was a pilot RCT ^34^, while the other was a mixed-methods pilot study ^41^.

In a pilot time-cluster RCT, Giraud *et al.* tested the effect of introducing structured mirror usage into post-operative recovery in elderly ICU patients (≥70 years) after cardiac surgery ^34^. Mirror usage was standardized by developing a protocol for nurses and physiotherapists, aiming to use both small, personal mirrors as well as larger posture mirrors in order to help the patient with reorientation and self-awareness, enhance multisensory feedback on minor procedures, and augment passive and active physical therapies. The control cohort received usual care, including allowing control patients who brought a mirror from home to use it per their normal habits. After comparing the usual care group with the mirrors group, no significant improvement was found in delirium incidence, ICU days with delirium, or the proportion of the total ICU length of stay that the patient spent delirious ( Table 3 & Table 4).

The mixed-methods study by Lisann-Goldman *et al.* had subjects who were 40 years of age or older participate in Langerian mindfulness discussion exercises both prior to and after elective cardiac surgery with cardiopulmonary bypass ^41^. In addition to discussion exercises, patients listened to an audio file before surgery. This audio file walked them through techniques on how to re-assess one’s situation and improve their outlook by taking emotional control of the situation, encouraging the patients to focus on the process of change and allowing oneself to accept new ideas and remain confident about the unknown. The discussion exercises continued post-operatively twice daily. In contrast, the ‘informational control’ group went through normal pre-operative discussions followed by an audio file describing the process of cardiac surgery. They found that no subject developed delirium in either the interventional or the ‘informational control’ group so the effectiveness of the treatment could not be assessed.

### Bundled protocols

***‘Wake Up and Breathe’ protocol***. Khan *et al.* designed a ‘Wake Up and Breathe’ protocol in a pre-post interventional study to assess for any change in delirium and sedation in mechanically ventilated, adult ICU patients ^39^. They modified elements of the Awakening and Breathing Controlled trial (ABC) to implement a spontaneous awakening trial and daily sedation vacation followed by a spontaneous breathing trial, depending on the patient’s response ^51^. Delirium was assessed by CAM-ICU, and both the incidence and prevalence of delirium were analyzed, with the study finding no significant change in either measured outcome ( Table 3).

***ABCDE(F) bundles***. Five of the 15 studies which examined delirium bundles studied the effectiveness of ABCDE(F) bundle protocols on reducing delirium. ABCDE(F) bundles have multiple components including: spontaneous awakening (A) and breathing (B) trials, interdisciplinary coordination of sedatives and medications (C), delirium screening and management (D), early mobilization (E), and family engagement and involvement (F) ^52^. Of these five studies, two were pre-post studies ^26, 40^, one was a prospective multicenter cohort study ^37^, one was a quasi-experimental quality improvement project ^30^, and one was a retrospective cohort study ^27^. Three measured delirium outcomes using CAM-ICU ^26, 30, 40^, one utilized ICDSC ^27^, and one multicenter study used either CAM-ICU or ICDSC ^37^.

Balas *et al.* assessed the impact of an ABCDE bundle on adult ICU patients, evaluating the prevalence and duration of delirium in both total days and percent of ICU days spent delirious, with a pre-post study ^26^. The prevalence and percent of ICU days spent delirious were improved in the post period with p-values of 0.03 and 0.003 respectively ( Table 3 & Table 4). However, the overall duration of delirium was not significantly different ( Table 4).

The retrospective assessment of an ABCDE bundle by Bounds *et al.* evaluated its effect on delirium prevalence and duration in an adult ICU population ^27^. Both the prevalence and duration were significantly decreased in the ABCDE bundle group (p= 0.01 and 0.001 respectively; ( Table 3 & Table 4).

Kram *et al.* also looked at a similar patient cohort, all adult patients 18 or older admitted to the ICU, in a pre-post ABCDE bundle study with a smaller subject population (Kram, n=83; Balas, n=296; Bounds, n=159) ^40^. They assessed the effectiveness of the ABCDE bundle on delirium by measuring delirium prevalence and comparing it to a control based on literature values. The measured delirium prevalence of 19% ( Table 3) fell outside their cited literature values of 20–80%.

Chai initiated an ABCDEF bundle in a mixed ICU setting and analyzed delirium incidence in the adult patients in a pre-post, quasi-experimental quality improvement project ^30^. Delirium incidence was compared between morning and night occurrences (morning 08:00–11:00; night 20:00–23:00); both showed significant improvement in the intervention group with a p-value <0.001 for both morning and evening measurements ( Table 3).

A prospective cohort study performed by Pun *et al.* through a national quality improvement initiative compared complete ABCDEF bundle performance with proportional ABCDEF bundle performance in adult ICU patients with an ICU stay of at least 48 consecutive hours ^37^. Complete bundle performance was defined as a patient-day where 100% of the eligible bundle elements were performed, whereas proportional performance was anything less ^37^. Their study was comprehensive, including 10,840 patients for delirium outcome analysis across 68 ICUs in the United States and Puerto Rico ^37^. When comparing the incidence of delirium between patients with complete and proportional ABCDEF bundle performance, they found that patients with complete performance were significantly less likely to develop delirium ( Table 3) ^37^. In an additional analysis, Pun *et al.* found a dose-dependent reduction of delirium incidence when the more eligible ABCDEF bundle elements were performed (p < 0.0001) ^37^. It is worth noting that this study had a high rate of ‘missingness’ for delirium data and the analysis team chose not to perform multiple imputations ^37^.

***Other bundled protocols***. The remaining nine bundle studies developed new, unique bundles. They included four pre-post studies ^28, 46, 49, 50^, three RCTs ^35, 36, 43^, one quasi-experimental study ^25^, and one action research study ^33^. Seven assessed delirium incidence and duration using CAM-ICU ^25, 28, 33, 35, 43, 49, 50^, one used NEECHAM ^36^, and one used ICDSC ^46^.

A quasi-experimental study designed by Arbabi *et al.* developed a multi-component delirium management bundle comprised of staff education and environmental and non-pharmacologic care changes ^25^. They measured the effectiveness of their bundle by assessing delirium incidence and duration in all adult patients admitted to the general ICU, finding a significant difference in both outcomes (p = 0.01 and 0.001 respectively; Table 3 & Table 4).

Bryczkowski *et al.* assessed the effectiveness of their bundle, which included a staff-patient-family education program, medication management strategies, and non-pharmacological sleep enhancement protocols, on delirium incidence and delirium free days in patients over the age of 50 years ^28^. The research team found no significant improvement in delirium incidence ( Table 3), although the average total number of delirium-free days out of 30 changed significantly from 24 to 27 between groups (p=0.002; Table 4).

Another bundle study developed by Fallahpoor *et al.* focused specifically on adults admitted to the ICU after elective CABG in an action research study. Their post-CABG delirium management bundle was assessed in an action research study and had three elements focusing on pre-, intra-, and post-operative methods to identify delirium risk factors, optimize time spent in surgery, and introduce staff education and post-operative environmental changes ^33^. Delirium related outcomes included the incidence ratio and total number of recorded delirium events, with significant differences found in both (p=0.001 and 0.008 respectively; Table 3).

In the RCT conducted by Guo *et al.*, the effect of a bundle consisting of cognitive prehabilitation, post-operative cognitive stimulation activities, environmental changes, music therapy, and non-pharmacologic care changes on delirium incidence and duration after oral tumor resection in patients aged 65–80 years was studied ^35^. The incidence of delirium improved significantly overall, but was only significantly different on post-operative day one compared to days two and three (p=0.035, p=0.374, p=0.364 respectively; Table 3); the duration of delirium also differed significantly (p< 0.001; Table 4).

Hamzehpour *et al.* designed an RCT and implemented the Roy adaptation nursing model for all adult ICU patients, which focuses on balance of nutrition, electrolytes, and fluids while promoting activity, sleep hygiene, and monitoring of circulation and endocrine function ^36^. Their primary delirium-specific outcomes were incidence and severity, and they analyzed both outcomes for two time points (morning & night) for seven days. Their research only showed significant improvements to incidence on day seven, both morning and night (p<0.008 and p<0.05; Table 3), but delirium severity, assessed with NEECHAM, improved through the morning of day four to the night of day seven at all measured time points (every time point, p≤0.028; Table 5).

Moon and Lee implemented a bundle which included early cognitive assessments and reorientation, sensory aids, environmental changes, consistent care staff and location, familiar items from home, nursing care changes, and early mobility as part of an RCT aimed at assessing delirium incidence in adult ICU patients with at least a 48 hour stay ^43^. Their study did not show a significant difference between the intervention and the control group who received usual care ( Table 3).

In a pre-post, observational quality improvement project, Rivosecchi *et al.* combined staff education with a non-pharmacologic bundle to look at incidence and duration of delirium in any adult patient aged 18 or older admitted to the medical ICU ^46^. Their M.O.R.E. bundle included (M)usic, (O)pening blinds, (R)eorientation and cognitive stimulation, and (E)ye and ear care. Both delirium incidence and duration were significantly impacted, with incidence decreasing from 15.7% to 9.4% and a reduction in duration from 16.1% of the ICU stay to 9.6% (p = 0.04 and <0.001 respectively; Table 3, Table 4).

Sullinger *et al.* enrolled adult surgical-trauma ICU patients with acute delirium in a pre-post retrospective study, tailoring their bundle to incorporate staff education with sensory aids, healing arts techniques, mobility, environmental changes, and family presence ^49^. Their bundle also included the initiation of anti-psychotic medications if non-pharmacologic tactics failed. The only specifically delirium related outcome was the number of days spent delirious, resulting in a significant decrease from 8.2 to 4.5 median days ( Table 4).

Any patient 18 years or older admitted to a cardiothoracic ICU after CABG surgery was analyzed for incidence, duration, and severity of delirium by Zhang *et al.* in a prospective pre-post study ^50^. Their delirium bundle targeted risk factor screening and modifications, including increased family visits, reorientation, and changes to nursing care. The only significant improvement was to incidence of delirium which dropped from 29.93% to 13.48% ( Table 3), while the intervention had no impact on duration or severity ( Table 4 & Table 5).

## Discussion

### Summary of findings

Our review included 27 trials that evaluated the effect of various non-pharmacological treatment and management protocols on delirium in an ICU setting. Assessment of the efficacy of these protocols in the last five years was most commonly done by considering incidence and/or prevalence. A total of 25 studies assessed for the effects of these protocols on incidence and/or prevalence, with 11 studying individual approaches and 14 studying bundles. Of these 25 trials, 11 reported significant improvements overall ^24– 27, 30, 32, 33, 37, 46, 47, 50^, nine found no significant improvement ^28, 29, 31, 34, 38, 39, 43, 45, 48^, and two only found significant change at certain time points ^35, 36^; the remaining three did not analyze for statistical significance of their results ^40– 42^. The 11 effective interventions for incidence and/or prevalence were primarily bundled protocols (eight trials) ^25– 27, 30, 33, 37, 46, 50^, followed by family approaches (two trials) ^32, 47^, and early and intensive OT (one trial) ^24^. The two studies with time point dependent changes were both bundles, one non-pharmacologic ^35^ and the other introduced the Roy adaptation nursing model ^36^. The multicomponent non-pharmacologic bundle found improvements in incidence both overall and on day one, while the Roy adaptation trial only saw a change in incidence on day seven in both the morning and the evening. Three studies did not analyze for statistical significance ^40– 42^; however, the family caregiver intervention saw an overall reduction in the percent of subjects who developed delirium from 71.40% to 43.80% ^42^. The study on mindfulness exercises had no subjects in either investigational group develop delirium ^41^, and an ABCDE bundle reported a post-bundle incidence of 19% but stated there was no pre-bundle data with which to compare ^40^. No other studies that looked at delirium incidence were effective.

In addition to incidence and prevalence, another common outcome was a change in the duration of delirium. Sixteen of the reviewed studies evaluated the duration of delirium, eight focusing on individual interventions and eight introducing bundled protocols. Of these 16 studies, eight found significant changes overall ^24, 25, 27, 28, 35, 45, 46, 49^, two had significant improvements at select time points ^26, 44^, and five did not have significant results ^29, 34, 38, 48, 50^; the one remaining study did not assess for statistical significance ^42^. Six of the eight successful trials were bundles and both of the two effective individual therapies were mobility-focused (early and intensive OT, and FES) ^24, 45^. Only four studies looked at delirium severity ^24, 36, 42, 50^ with only one finding any significant results, and only finding them at select time-points (Roy Adaptation Model) ^36^.

The pilot RCT performed by Alvarez *et al.* utilized a unique method for assessing the performance of their intervention. In addition to assessing delirium incidence, they measured the ratio of delirium duration to the amount of time exposed to the treatment (IRR) ^24^. They found that IRR decreased as the time exposed to treatment increased to a significant degree (p= 0.000). This ratio could be explained in three ways. Either the duration of delirium stayed the same as the time exposed to treatment increased, the duration of delirium increased slower than the time exposed increased, or the duration of delirium decreased while the time of exposure increased. However, the last explanation is impossible, due to the duration of delirium being a sum overtime which could not decrease, such that the result must be explained by either a small increase or no increase in the duration of delirium. If the decrease in the IRR is explained by smaller and smaller increases to delirium duration it is likely that the IRR results from either the trend of patients slowly becoming healthier over time, or the conjunction of that with the intervention. However, if the IRR is explained by the delirium duration ceasing to increase, then once it stops the treatment may still be effective, but it is not becoming more effective over time and plateaus in effectiveness.

### Implications of results and application to practice

The reviewed studies focused on individual interventions that had a wide range of limitations and were, on the whole, less effective than bundled protocols in the treatment and management of delirium. Many of these studies had limited reliability due to small or extremely small sample sizes ^29, 41, 44, 45^. Additionally, even when results were significant, they often had limited applications to practice due to the prevalence of restricted populations. Three of the individual intervention studies limited their study cohort to elderly adults ^15, 24, 38^, a population which is at an increased risk of delirium. It is unclear whether these results would apply to younger patients. Studied populations were also commonly narrowed to either exclusively intubated patients ^45^, non-intubated patients ^24, 32^ or patients with a particular illness ^34, 42, 45^. Another possible limitation was in the questionable reliability of delirium assessment criteria. This is mentioned by Campbell who stated that 35% of CAM-ICU were incorrectly labeled as ‘unable to assess’ ^29^. The question of reliability was also raised in Lisann-Goldman *et al.*. This study could not assess the effectiveness of their intervention as no patients developed delirium ^41^. However, this could be explained by the fact that fully sedated patients were considered ‘not delirious’ since CAM-ICU could not be performed. The authors also noted that since it often takes weeks or months to fully integrate new behavioral thought techniques, a study focused on changing thought patterns in days would not entirely reflect the full benefit if any were present ^41^.

Eight of the individual intervention studies were RCTs. This type of study introduces the possibility of additional limitations due to the nature of its design. Two of these RCTs had a high risk of bias ^32, 44^ due to a failure to blind patients and personnel, as well as blinding of the outcome assessment and improper allocation concealment. The question of blinding raises another possible limitation of many of these studies, namely the possibility of the Hawthorne effect in patients who knew that they were being observed and receiving an intervention for the treatment/prevention of delirium.

The 15 studies which investigated bundled protocols had, overall, larger sample sizes, fewer cohorts with limited populations, and indicated better reliability in their delirium assessment than the studies which focused on individual interventions. The smallest sample size was 83 patients ^40^; however, this study did not split the sample into multiple cohorts and all patients received the intervention. One study had a sample size of 89 ^49^, and all other studies had a sample size of at least 100 patients. A total of five studies restricted their studied population beyond adult ICU patients ^28, 33, 35, 39, 50^. Two of these limited their population by age ^28, 35^; however, Bryczkowski *et al.*, despite limiting their population by age, included any patients greater than 50 years old, younger than the age when delirium risk is noted to increase ^53, 54^. One study limited its population to mechanically ventilated patients ^39^, two considered only patients undergoing CABG ^33, 50^, and one studied patients after oral tumor resection ^35^. These population restrictions could limit the generalizability and applicability of the interventions; however, this risk is reduced since bundles were often investigated in multiple studies with similar results.

Only three of the bundle studies were RCTs ^35, 36, 43^. Each of these RCTs had an unclear risk of bias with the most common risk being the inability to blind participants and personnel. The impossibility of blinding in delirium intervention studies makes RCTs a questionable approach. Eight of the bundle studies, recognizing blinding as an impossibility, chose to conduct pre-post prospective studies rather than RCTs ^26, 28, 30, 39, 40, 46, 49, 50^. These studies carried a lower risk of introducing bias to their studies and avoided crossover between arms. The pre-post study performed by Kram *et al.* had the major limitation of not including a pre cohort and only comparing the results of their intervention with literature values ^40^. Additionally, while they found their measured delirium prevalence to fall outside their included literature values (19%), this prevalence falls within the values provided by the current literature (19- 87%) ^2^. Chai’s pre-post study had a delirium assessment with questionable reliability. While all other studies assessed delirium whenever the Richmond Agitation-Sedation Scale (RASS) was ≥ -3, they reported that patients were unable to be assessed whenever RASS was < -2, resulting in a greater proportion of patients not assessed for delirium ^30^.

Given the findings of this systematic review, further research is warranted in order to confirm these results and apply them to other patient populations. Multicomponent, bundled approaches were more successful at improving delirium outcomes compared to individual techniques; however, the effective individual tactic of family engagement was included as a component in the effective bundles. Although a majority of the reviewed bundles were effective, it is difficult to compare results as the trials had large differences in study design, enrollment numbers, and delirium assessment measures.

### Strengths and limitations

The strengths of our systematic review include thorough search terms and the methodology to assess a vast majority of recent literature in this field.

One limitation of this systematic review is that we only focused on trials within the past five years which excluded some well-cited early studies on delirium. We also did not evaluate other listed outcomes which could provide additional insight into any change in delirium status. Since the condition can be transient and delirium screenings are not performed as frequently throughout the ICU day as other measurements, outcomes such as restraint use or amount of prescribed sedatives or anti-psychotic medications would be beneficial to assess in this setting. While the decision to omit exclusion criteria on study design allowed for assessment of a broader range of trials, it was difficult to compare outcomes when multiple differing designs and measurement tools were used. Although the CAM-ICU was widely used, some studies used alternative tools and there was no standardized way of defining or measuring delirium duration or severity. A different measurement tool was used to evaluate severity in each of the four studies reviewing this outcome, and duration was defined in a multitude of fashions. Combining this realization with the fact that some studies focused on highly specific subpopulations suggests that some trials may need to be replicated in a standardized fashion to account for any differences in methodology or subjective assessments.

## Conclusions

Many ICU delirium treatment and management protocols were developed and tested within the last five years in a variety of study designs. Few trials on individual interventions had positive effects on delirium incidence and duration, but multicomponent bundles were found to be more effective overall while incorporating the effective individual intervention of family engagement. Based on the results of bundle studies, the implementation of multi-component protocols in ICUs can reduce ICU delirium, thereby reducing cost of care, improving overall outcomes, and limiting time spent mechanically ventilated, medicated, or admitted. Despite these results, further research is needed on individual interventions in order to improve specific elements of multicomponent bundles by adding or removing ineffective therapies. Additional research is also warranted to evaluate for any positive effects in more generalized hospital populations.

## Data availability

### Underlying data

All data underlying the results are available as part of the article and no additional source data are required.

### Extended data

OSF: The Effect of Non-Pharmacologic Strategies on Prevention or Management of Intensive Care Unit Delirium: A Systematic Review. https://doi.org/10.17605/OSF.IO/Y7NQ6 ^20^.

Registration DOI: 10.17605/OSF.IO/C3RHF

This project contains the following extended data:

- Supplementary Appendix 2 – Database search terms
- Supplementary Table 1 – Data extraction form
- Supplementary Table 2 – Risk of bias assessment

Data are available under the terms of the ‘Creative Commons Zero "No rights reserved" data waiver’ (CC0 1.0 Public domain dedication).

### Reporting guidelines

OSF: PRISMA checklist for ‘The Effect of Non-Pharmacologic Strategies on Prevention or Management of Intensive Care Unit Delirium: A Systematic Review’. https://doi.org/10.17605/OSF.IO/Y7NQ6 ^20^

Registration DOI: 10.17605/OSF.IO/C3RHF

Data are available under the terms of the ‘Creative Commons Zero "No rights reserved" data waiver’ (CC0 1.0 Public domain dedication).
