# Supplementary material for: The effect of non-pharmacologic strategies on prevention or management of intensive care unit delirium: a systematic review
Source: F1000Res. 2022 Jun 24;9:1178. Originally published 2020 Sep 28. [Version 3] doi: 10.12688/f1000research.25769.3 (PMC9449425; doi:10.12688/f1000research.25769.3)
Supplement: Peer review history

# Peer review: Reviewer response for version 3

**DOI:** 10.5256/f1000research.134761.r142132

**Published:** 2022-09-06

**Authors:** Claudia D. Spies, Henning Krampe, Fatima Yürek

**Affiliations:** 1 Department of Anesthesiology and Operative Intensive Care Medicine (CCM, CVK), Charité - Universitätsmedizin Berlin, Berlin, Germany; 2 Charité - Universitätsmedizin Berlin, Berlin, Germany

We would like to thank the authors for the thorough response to our review, as well as for addressing all relevant concerns. The authors did a very good job with the revision, so that the manuscript has improved very much. We think the revisions add much clarity to the paper, which will be of much interest and use for readers.

Are the rationale for, and objectives of, the Systematic Review clearly stated?

Yes

Is the statistical analysis and its interpretation appropriate?

Partly

Are sufficient details of the methods and analysis provided to allow replication by others?

Yes

Are the conclusions drawn adequately supported by the results presented in the review?

Partly

Reviewer Expertise:

NA

We confirm that we have read this submission and believe that we have an appropriate level of expertise to confirm that it is of an acceptable scientific standard.

# Peer review: Reviewer response for version 2

**DOI:** 10.5256/f1000research.30705.r74838

**Published:** 2022-05-18

**Authors:** Claudia D. Spies, Fatima Yürek, Alawi Luetz

**Affiliations:** 1 Department of Anesthesiology and Operative Intensive Care Medicine (CCM, CVK), Charité - Universitätsmedizin Berlin, Berlin, Germany; 2 Charité - Universitätsmedizin Berlin, Berlin, Germany

**Major issues:**

1. The evaluations do not clearly differentiate between ICU delirium in critically ill patients and postoperative delirium in the ICU. The risk factors for critically ill patients with acute organ failure requiring ICU treatment and patients who have had elective or emergency surgery may differ. It would be better if the populations of critically ill patients were considered separately from the population of elective and emergency surgery patients. Given the predisposing and precipitating risk factors that are additional to surgery, this could impact the effectiveness of nonpharmacologic preventive measures.

**Minor issues:**

1. The effectiveness and efficiency of non-pharmacological measures is limited to the intensive care unit. It remains open how non-pharmacological measures affect patients in the normal ward or to what extent the results can be transferred to structures in the normal ward. The effect of mobility in the normal ward offers a much greater advantage for the patients (preservation of authority, self-care, and independence) than it could be shown in the ICU. Therefore, this should be emphasized more clearly.
2. The age distribution in the studies considered is very broad. The question arises whether the integration of relatives into the treatment concept of non-pharmacological preventive measures for delirium has shown an independent preventive effect or whether this effect could be shown specifically in certain age groups.

Are the rationale for, and objectives of, the Systematic Review clearly stated?

Yes

Is the statistical analysis and its interpretation appropriate?

Partly

Are sufficient details of the methods and analysis provided to allow replication by others?

Yes

Are the conclusions drawn adequately supported by the results presented in the review?

Partly

Reviewer Expertise:

NA

We confirm that we have read this submission and believe that we have an appropriate level of expertise to confirm that it is of an acceptable scientific standard, however we have significant reservations, as outlined above.

# Response

**Published:** 2022-06-03

**Authors:** Azra Bihorac

**Affiliations:** Department of Anesthesiology, University of Florida College of Medicine, USA

Response to Major Issue #1:  Thank you so much for pointing this out – this distinction is imperative. We added a brief segment to the limitations portion of our discussion section discussing this important delineation.

 Response to Minor Issue #1:  This is a great consideration, thank you for mentioning this limitation. We added related information to the future directions portion in the article’s discussion section.

 Response to Minor Issue #2:  Thank you for also bringing this distinction to our attention. This is a valid concern – certain strategies may be more effective for older versus younger ICU delirium patients and these sub-populations may have different underlying risk factors and etiologies. We added a segment into our discussion section regarding this aspect.

# Peer review: Reviewer response for version 1

**DOI:** 10.5256/f1000research.28439.r73595

**Published:** 2020-11-05

**Authors:** Elizabeth Mahanna-Gabrielli

**Affiliations:** 1 Department of Anesthesiology, Perioperative and Pain Medicine, University of Miami Miller School of Medicine, Miami, FL, USA

This manuscript is a systematic review of the effect of non-pharmacologic strategies on prevention or management of delirium in the ICU. The manuscript is comprehensive, well written, and follows appropriate PICOS framework and PRISMA guidelines for a systematic review.

 The introduction is well written, succinct and appropriately gives background information on the importance of ICU delirium prevention and management.

 The methods clearly state how the authors followed the PRISMA guidelines. The authors searched Pubmed, Embase, Cochrane Central, Web of Science, CINAHL, and Clinciattrials.gov. MeSH terms appropriately included delirium and other terms that are often used to describe the delirium syndrome such as “acute brain dysfunction” and “psychosis”. A full list of the search terms for each database is included in supplementary appendix 2 to allow for reproducibility.

1. Could the authors clarify if the date range was 5 years from the date of search (Jan 1, 2014 to May 15, 2019) as stated in the manuscript? Or was it 10 years, as is listed in the search terms of the supplementary appendix 2?
2. Did all the authors screen the titles and abstracts? And did all the authors independently review the full text of the 113 eligible articles, or only some of the authors?
  Bias was appropriately assessed by the Cochrane risk of bias tool. Disagreements between authors were adjudicated as a group through discussion.
  The results are clearly written and concise.
3. For Table 2, I found it confusing using x’s, y’s and o’s. It may be better to title the Table “Summary of delirium outcomes by statistical significance (p<0.05),” and then spell out “yes” instead of y, and either “no” or “not significant” instead of x. For the outcomes that were measured but not analyzed, it may be clearer to write “not analyzed” or “NA” rather than “o.”

The other tables were clear and appropriately organized. 

 The discussion is well written and discusses the limitations of the included studies and of this systematic review. I have no suggestions for improvement.

Are the rationale for, and objectives of, the Systematic Review clearly stated?

Yes

Is the statistical analysis and its interpretation appropriate?

Yes

Are sufficient details of the methods and analysis provided to allow replication by others?

Yes

Are the conclusions drawn adequately supported by the results presented in the review?

Yes

Reviewer Expertise:

perioperative brain health, delirium - ICU and postoperative, sleep

I confirm that I have read this submission and believe that I have an appropriate level of expertise to confirm that it is of an acceptable scientific standard.

# Response

**Published:** 2021-06-16

**Authors:** Azra Bihorac

**Affiliations:** Department of Anesthesiology, University of Florida College of Medicine, USA

1. **Could the authors clarify if the date range was 5 years from the date of search (Jan 1, 2014 to May 15, 2019) as stated in the manuscript? Or was it 10 years, as is listed in the search terms of the supplementary appendix 2? **Thank you so much for this comment so that we can clarify. The searches for each database listed in Supplementary Appendix 2 were initially performed for 10 years from the date of the search. During the title and abstract screening process, we ultimately decided to narrow the results further to within 5 years of the dated search. This step can be seen in Fig 1 PRISMA Record Screening Flow Chart, where we removed 3039 records prior to 2014. We added a statement to our methods section to further clarify this aspect.
2. **Did all the authors screen the titles and abstracts? And did all the authors independently review the full text of the 113 eligible articles, or only some of the authors?** ****Thank you for informing us of this insufficiency. Four authors (JSC, HH, MMR, RB) screened the titles and abstracts, and three authors independently reviewed the full text (JSC, HH, JL). We added a statement to our methods section clarifying this point.
3. **For Table 2, I found it confusing using x’s, y’s and o’s. It may be better to title the Table “Summary of delirium outcomes by statistical significance (p<0.05),” and then spell out “yes” instead of y, and either “no” or “not significant” instead of x. For the outcomes that were measured but not analyzed, it may be clearer to write “not analyzed” or “NA” rather than “o.” **Thank you for the feedback about this table. This change will definitely make our table more clear to the reader, and Table 2 has been updated to reflect this amendment.
